# (*E*)-2-(2,4-Dihydroxy­benzyl­ideneamino)benzonitrile

**DOI:** 10.1107/S1600536809020182

**Published:** 2009-06-06

**Authors:** Ting Liu

**Affiliations:** a Biology and Chemistry Department, Nanchang University College of Science and Technology, Nanchang 330029, People’s Republic of China

## Abstract

The mol­ecule of the title compound, C_14_H_10_N_2_O_2_, adopts the phenol–imine tautomeric form. The dihedral angle between the planes of the two benzene rings is 13.84 (13)°. A strong intra­molecular O—H⋯N hydrogen-bonding inter­action stabilizes the mol­ecular conformation. In the crystal structure, centrosymmetrically related mol­ecules are linked into dimers by inter­molecular C—H⋯O and O—H⋯N hydrogen bonds.

## Related literature

For the crystal structures of related compounds, see: Cheng *et al.* (2006 ▶); Xia *et al.* (2008 ▶). For bond-length data, see: Allen *et al.* (1987 ▶).
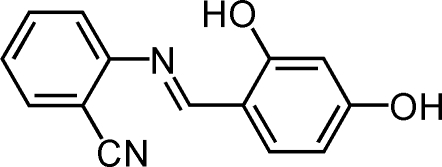


## Experimental

### 

#### Crystal data

C_14_H_10_N_2_O_2_
*M* *_r_* = 238.24
Monoclinic, 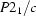

*a* = 13.322 (3) Å
*b* = 5.7505 (12) Å
*c* = 16.132 (3) Å
β = 108.97 (3)°
*V* = 1168.7 (5) Å^3^
*Z* = 4
Mo *K*α radiation
μ = 0.09 mm^−1^
*T* = 293 K
0.20 × 0.20 × 0.20 mm

#### Data collection

Rigaku SCXmini diffractometer
Absorption correction: multi-scan (*CrystalClear*; Rigaku, 2005 ▶) *T*_min_ = 0.973, *T*_max_ = 0.979
11536 measured reflections
2683 independent reflections
1394 reflections with *I* > 2σ(*I*)
*R*_int_ = 0.073

#### Refinement

*R*[*F* ^2^ > 2σ(*F* ^2^)] = 0.059
*wR*(*F* ^2^) = 0.153
*S* = 1.01
2683 reflections
167 parameters
H atoms treated by a mixture of independent and constrained refinement
Δρ_max_ = 0.15 e Å^−3^
Δρ_min_ = −0.16 e Å^−3^

### 

Data collection: *CrystalClear* (Rigaku, 2005 ▶); cell refinement: *CrystalClear*; data reduction: *CrystalClear*; program(s) used to solve structure: *SHELXS97* (Sheldrick, 2008 ▶); program(s) used to refine structure: *SHELXL97* (Sheldrick, 2008 ▶); molecular graphics: *SHELXTL* (Sheldrick, 2008 ▶); software used to prepare material for publication: *SHELXL97*.

## Supplementary Material

Crystal structure: contains datablocks I, global. DOI: 10.1107/S1600536809020182/rz2327sup1.cif

Structure factors: contains datablocks I. DOI: 10.1107/S1600536809020182/rz2327Isup2.hkl

Additional supplementary materials:  crystallographic information; 3D view; checkCIF report

## Figures and Tables

**Table 1:** Hydrogen-bond geometry (Å, °)

*D*—H⋯*A*	*D*—H	H⋯*A*	*D*⋯*A*	*D*—H⋯*A*
O1—H1*A*⋯N2	0.95 (3)	1.70 (3)	2.581 (2)	152 (3)
O2—H2*A*⋯N1^i^	0.82	2.03	2.835 (3)	166
C11—H11*A*⋯O1^i^	0.93	2.56	3.386 (3)	148

Symmetry code: (i) 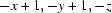.

**Fig. 1.**
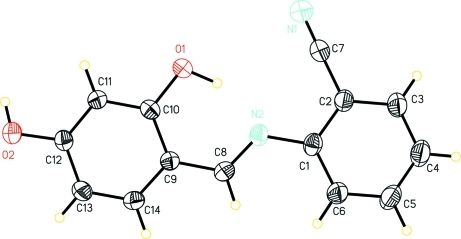
The molecular structure of the title compound, showing the atomic numbering scheme. Displacement ellipsoids are drawn at the 30% probability level.

**Fig. 2.**
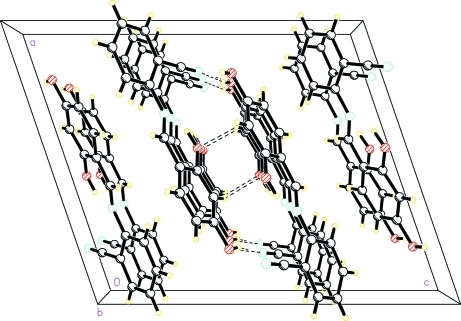
Packing diagram of the title compound, showing the structure along the b axis. Hydrogen bonds are shown as dashed lines.

## supplementary crystallographic information

### Comment

Schiff base compounds have attracted great attention and have been extensively investigated due to their important role in the development of coordination chemistry related to catalysis and enzymatic reactions, magnetism and molecular architectures. Herein, the synthesis and crystal structure of the title compound is reported.

The molecular structure of the title compound is shown in Fig. 1. The Schiff-base molecule adopts a non-planar conformation, with the dihedral angle between the two aromatic rings of 13.84 (13)°, and displays a *trans* configuration with respect to the C8=N2 double bond. Bond lengths (Allen *et al.*, 1987) and angles are normal and in good agreement with those reported for 5-chloro-2-(2-hydroxybenzylideneamino)benzonitrile (Cheng *et al.*, 2006) and 2-(2-hydroxybenzylideneamino)benzonitrile (Xia *et al.*, 2008). There is an strong intramolecular O—H···N hydrogen bond stabilizing the molecular conformation (Table 1). In the crystal structure (Fig. 2), centrosymmetrically related molecules are linked into dimers by intermolecular C—H···O and O—H···N hydrogen bonds (Table 1).

### Experimental

The title compound was prepared by refluxing a mixture of 2,4-dihydroxybenzaldehyde (0.552 g, 4 mmol) and 2-aminobenzonitrile (0.472 g, 4 mmol) in ethanol (20 ml). The reaction mixture was refluxed for 5 h under stirring, then cooled to room temperatureand and the resulting yellow precipitate was filtered off. Crystals of the title compound suitable for X-ray analysis were obtained by slow evaporation of an ethanol solution.

### Refinement

The H bound to O1 was located in a difference Fourier map and refined freely. All other H atoms were located geometrically and treated as riding atoms, with O—H= 0.82 Å, C—H = 0.93–0.97 Å, and with *U*iso(H) = 1.2*U*eq(C) or 1.5*U*eq(O).

### Crystal data

**Table:**

C_14_H_10_N_2_O_2_	*F*(000) = 496
*M**_r_* = 238.24	*D*_x_ = 1.354 Mg m^−^^3^
Monoclinic, *P*2_1_/*c*	Mo *K*α radiation, λ = 0.71073 Å
Hall symbol: -P 2ybc	Cell parameters from 8059 reflections
*a* = 13.322 (3) Å	θ = 3.1–27.8°
*b* = 5.7505 (12) Å	µ = 0.09 mm^−^^1^
*c* = 16.132 (3) Å	*T* = 293 K
β = 108.97 (3)°	Prism, yellow
*V* = 1168.7 (5) Å^3^	0.20 × 0.20 × 0.20 mm
*Z* = 4	

### Data collection

**Table:**

Rigaku SCXmini diffractometer	2683 independent reflections
Radiation source: fine-focus sealed tube	1394 reflections with *I* > 2σ(*I*)
graphite	*R*_int_ = 0.073
Detector resolution: 13.6612 pixels mm^-1^	θ_max_ = 27.5°, θ_min_ = 3.2°
ω scans	*h* = −17→17
Absorption correction: multi-scan (*CrystalClear*; Rigaku, 2005)	*k* = −7→7
*T*_min_ = 0.973, *T*_max_ = 0.979	*l* = −20→20
11536 measured reflections	

### Refinement

**Table:**

Refinement on *F*^2^	Primary atom site location: structure-invariant direct methods
Least-squares matrix: full	Secondary atom site location: difference Fourier map
*R*[*F*^2^ > 2σ(*F*^2^)] = 0.059	Hydrogen site location: inferred from neighbouring sites
*wR*(*F*^2^) = 0.153	H atoms treated by a mixture of independent and constrained refinement
*S* = 1.01	*w* = 1/[σ^2^(*F*_o_^2^) + (0.0648*P*)^2^] where *P* = (*F*_o_^2^ + 2*F*_c_^2^)/3
2683 reflections	(Δ/σ)_max_ < 0.001
167 parameters	Δρ_max_ = 0.15 e Å^−^^3^
0 restraints	Δρ_min_ = −0.16 e Å^−^^3^

### Special details

**Table:**

Geometry. All e.s.d.'s (except the e.s.d. in the dihedral angle between two l.s. planes) are estimated using the full covariance matrix. The cell e.s.d.'s are taken into account individually in the estimation of e.s.d.'s in distances, angles and torsion angles; correlations between e.s.d.'s in cell parameters are only used when they are defined by crystal symmetry. An approximate (isotropic) treatment of cell e.s.d.'s is used for estimating e.s.d.'s involving l.s. planes.
Refinement. Refinement of *F*^2^ against ALL reflections. The weighted *R*-factor *wR* and goodness of fit *S* are based on *F*^2^, conventional *R*-factors *R* are based on *F*, with *F* set to zero for negative *F*^2^. The threshold expression of *F*^2^ > σ(*F*^2^) is used only for calculating *R*-factors(gt) *etc*. and is not relevant to the choice of reflections for refinement. *R*-factors based on *F*^2^ are statistically about twice as large as those based on *F*, and *R*- factors based on ALL data will be even larger.

### Fractional atomic coordinates and isotropic or equivalent isotropic displacement parameters (Å^2^)

**Table:**

	*x*	*y*	*z*	*U*_iso_*/*U*_eq_	
O2	0.79346 (11)	0.8933 (3)	0.08149 (11)	0.0566 (5)	
H2A	0.7911	0.7663	0.0578	0.085*	
N2	0.33856 (14)	0.9483 (3)	0.12987 (11)	0.0458 (5)	
O1	0.45029 (14)	0.6553 (3)	0.07575 (12)	0.0620 (5)	
C8	0.41862 (18)	1.0886 (4)	0.14982 (14)	0.0467 (6)	
H8A	0.4127	1.2327	0.1740	0.056*	
C9	0.51588 (16)	1.0290 (4)	0.13590 (13)	0.0413 (5)	
C12	0.70129 (17)	0.9304 (4)	0.09842 (14)	0.0430 (6)	
C5	0.1263 (2)	1.2217 (5)	0.20046 (18)	0.0709 (8)	
H5A	0.1164	1.3435	0.2349	0.085*	
C3	0.05604 (19)	0.8978 (5)	0.10712 (18)	0.0643 (7)	
H3A	−0.0004	0.8009	0.0784	0.077*	
C1	0.24136 (18)	1.0047 (4)	0.14176 (14)	0.0466 (6)	
C10	0.52810 (17)	0.8164 (4)	0.09675 (14)	0.0434 (6)	
C14	0.60082 (18)	1.1854 (4)	0.15679 (15)	0.0506 (6)	
H14A	0.5947	1.3258	0.1834	0.061*	
C11	0.61972 (16)	0.7695 (4)	0.07767 (14)	0.0447 (6)	
H11A	0.6266	0.6300	0.0509	0.054*	
C13	0.69260 (18)	1.1384 (4)	0.13929 (15)	0.0514 (6)	
H13A	0.7484	1.2443	0.1545	0.062*	
C2	0.15547 (18)	0.8609 (4)	0.09949 (15)	0.0500 (6)	
C7	0.17047 (19)	0.6737 (5)	0.04576 (18)	0.0575 (7)	
N1	0.18114 (18)	0.5237 (4)	0.00325 (17)	0.0777 (8)	
C6	0.22459 (19)	1.1855 (5)	0.19264 (16)	0.0598 (7)	
H6A	0.2806	1.2832	0.2218	0.072*	
C4	0.0418 (2)	1.0796 (5)	0.15775 (18)	0.0707 (8)	
H4A	−0.0247	1.1065	0.1632	0.085*	
H1A	0.392 (2)	0.729 (5)	0.0876 (17)	0.090 (10)*	

### Atomic displacement parameters (Å^2^)

**Table:**

	*U*^11^	*U*^22^	*U*^33^	*U*^12^	*U*^13^	*U*^23^
O2	0.0453 (10)	0.0602 (11)	0.0695 (12)	−0.0039 (8)	0.0261 (8)	−0.0088 (8)
N2	0.0384 (10)	0.0558 (12)	0.0429 (11)	0.0041 (10)	0.0130 (8)	−0.0002 (9)
O1	0.0445 (10)	0.0547 (11)	0.0895 (14)	−0.0107 (9)	0.0256 (9)	−0.0253 (9)
C8	0.0512 (14)	0.0476 (14)	0.0432 (14)	0.0018 (12)	0.0179 (11)	−0.0039 (10)
C9	0.0415 (13)	0.0418 (13)	0.0405 (13)	−0.0001 (11)	0.0133 (10)	−0.0021 (10)
C12	0.0384 (12)	0.0508 (14)	0.0401 (13)	−0.0001 (11)	0.0131 (10)	0.0016 (10)
C5	0.0568 (16)	0.096 (2)	0.0643 (18)	0.0081 (17)	0.0256 (14)	−0.0210 (16)
C3	0.0468 (15)	0.0802 (19)	0.0698 (18)	−0.0065 (14)	0.0241 (13)	−0.0108 (15)
C1	0.0435 (13)	0.0595 (15)	0.0386 (13)	0.0060 (12)	0.0155 (10)	0.0039 (11)
C10	0.0397 (13)	0.0443 (13)	0.0429 (13)	−0.0042 (11)	0.0092 (10)	−0.0004 (10)
C14	0.0545 (15)	0.0418 (14)	0.0574 (15)	−0.0033 (12)	0.0209 (12)	−0.0087 (11)
C11	0.0422 (13)	0.0449 (13)	0.0468 (13)	0.0001 (11)	0.0142 (10)	−0.0082 (10)
C13	0.0472 (14)	0.0504 (15)	0.0598 (16)	−0.0117 (12)	0.0218 (12)	−0.0073 (12)
C2	0.0448 (14)	0.0572 (15)	0.0507 (15)	0.0011 (12)	0.0192 (11)	−0.0018 (12)
C7	0.0501 (15)	0.0586 (17)	0.0681 (18)	−0.0079 (13)	0.0252 (13)	−0.0053 (14)
N1	0.0735 (17)	0.0691 (16)	0.0994 (19)	−0.0109 (14)	0.0406 (14)	−0.0236 (15)
C6	0.0499 (15)	0.0781 (19)	0.0521 (15)	−0.0044 (14)	0.0176 (12)	−0.0176 (14)
C4	0.0499 (16)	0.099 (2)	0.0692 (19)	0.0031 (16)	0.0278 (14)	−0.0109 (16)

### Geometric parameters (Å, °)

**Table:**

O2—C12	1.359 (2)	C3—C4	1.377 (3)
O2—H2A	0.8200	C3—C2	1.386 (3)
N2—C8	1.292 (3)	C3—H3A	0.9300
N2—C1	1.407 (3)	C1—C6	1.387 (3)
O1—C10	1.349 (3)	C1—C2	1.397 (3)
O1—H1A	0.95 (3)	C10—C11	1.379 (3)
C8—C9	1.427 (3)	C14—C13	1.368 (3)
C8—H8A	0.9300	C14—H14A	0.9300
C9—C14	1.398 (3)	C11—H11A	0.9300
C9—C10	1.409 (3)	C13—H13A	0.9300
C12—C11	1.383 (3)	C2—C7	1.436 (3)
C12—C13	1.389 (3)	C7—N1	1.139 (3)
C5—C6	1.372 (3)	C6—H6A	0.9300
C5—C4	1.380 (4)	C4—H4A	0.9300
C5—H5A	0.9300		
			
C12—O2—H2A	109.5	O1—C10—C9	121.1 (2)
C8—N2—C1	123.0 (2)	C11—C10—C9	120.6 (2)
C10—O1—H1A	104.5 (17)	C13—C14—C9	122.0 (2)
N2—C8—C9	122.0 (2)	C13—C14—H14A	119.0
N2—C8—H8A	119.0	C9—C14—H14A	119.0
C9—C8—H8A	119.0	C12—C11—C10	119.9 (2)
C14—C9—C10	117.6 (2)	C12—C11—H11A	120.1
C14—C9—C8	120.9 (2)	C10—C11—H11A	120.1
C10—C9—C8	121.5 (2)	C14—C13—C12	119.1 (2)
O2—C12—C11	122.5 (2)	C14—C13—H13A	120.5
O2—C12—C13	116.8 (2)	C12—C13—H13A	120.5
C11—C12—C13	120.7 (2)	C3—C2—C1	121.4 (2)
C6—C5—C4	120.8 (3)	C3—C2—C7	119.5 (2)
C6—C5—H5A	119.6	C1—C2—C7	119.1 (2)
C4—C5—H5A	119.6	N1—C7—C2	179.0 (3)
C4—C3—C2	119.3 (2)	C5—C6—C1	120.7 (2)
C4—C3—H3A	120.4	C5—C6—H6A	119.7
C2—C3—H3A	120.4	C1—C6—H6A	119.7
C6—C1—C2	117.9 (2)	C3—C4—C5	119.9 (2)
C6—C1—N2	125.9 (2)	C3—C4—H4A	120.1
C2—C1—N2	116.2 (2)	C5—C4—H4A	120.1
O1—C10—C11	118.2 (2)		

### Hydrogen-bond geometry (Å, °)

**Table:**

*D*—H···*A*	*D*—H	H···*A*	*D*···*A*	*D*—H···*A*
O1—H1A···N2	0.95 (3)	1.70 (3)	2.581 (2)	152 (3)
O2—H2A···N1^i^	0.82	2.03	2.835 (3)	166
C11—H11A···O1^i^	0.93	2.56	3.386 (3)	148

Symmetry codes: (i) −*x*+1, −*y*+1, −*z*.
